# Giant Cell Arteritis

**DOI:** 10.5334/jbsr.1487

**Published:** 2018-01-31

**Authors:** Bruno Coulier, Luc Montfort, Isabelle Gielen

**Affiliations:** 1 Clinique Saint-Luc, Bouge, BE; 2 Institute of Pathology and Genetics, Loverval, BE

**Keywords:** Giant cell arteritis, temporal arteritis, aortitis

## Case

**Figure 1:**
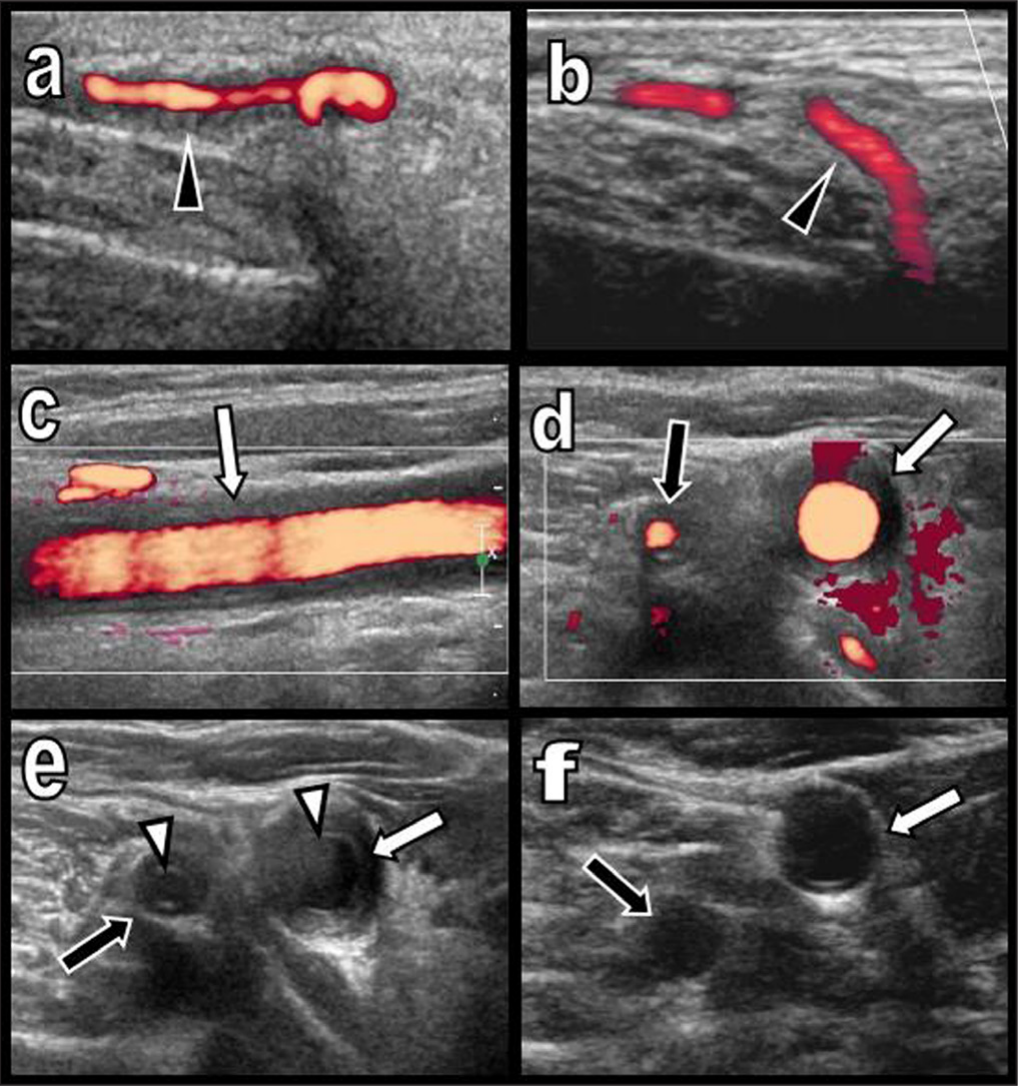
Colour Doppler Ultrasound of the temporal artery before **(a)** and after treatment **(b)** and of the right primitive carotid and vertebral arteries before **(c, d, e)** and after treatment **(f)**.

**Figure 2:**
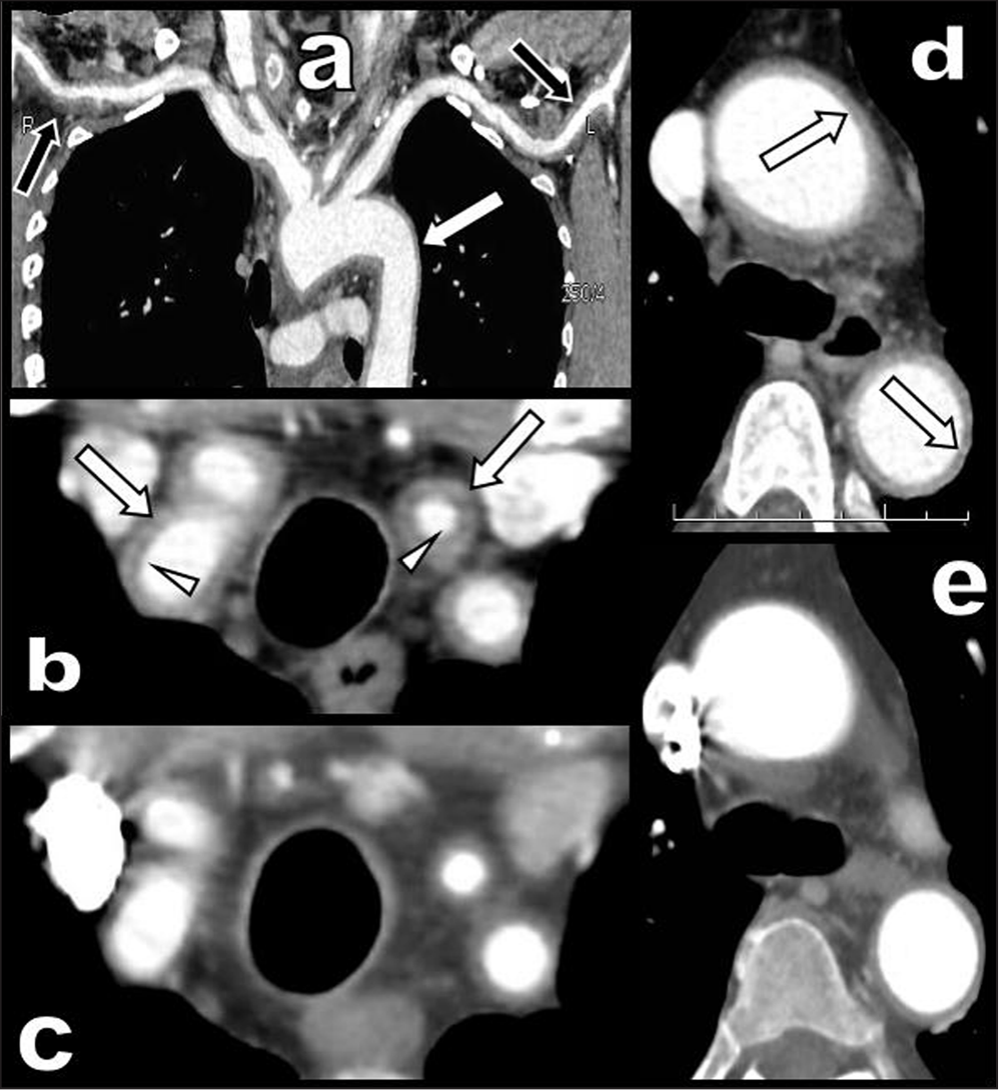
CT angiography of the aortic arch and of its large emerging arteries before **(a, b, d)** and after treatment **(c** and **e)**.

**Figure 3:**
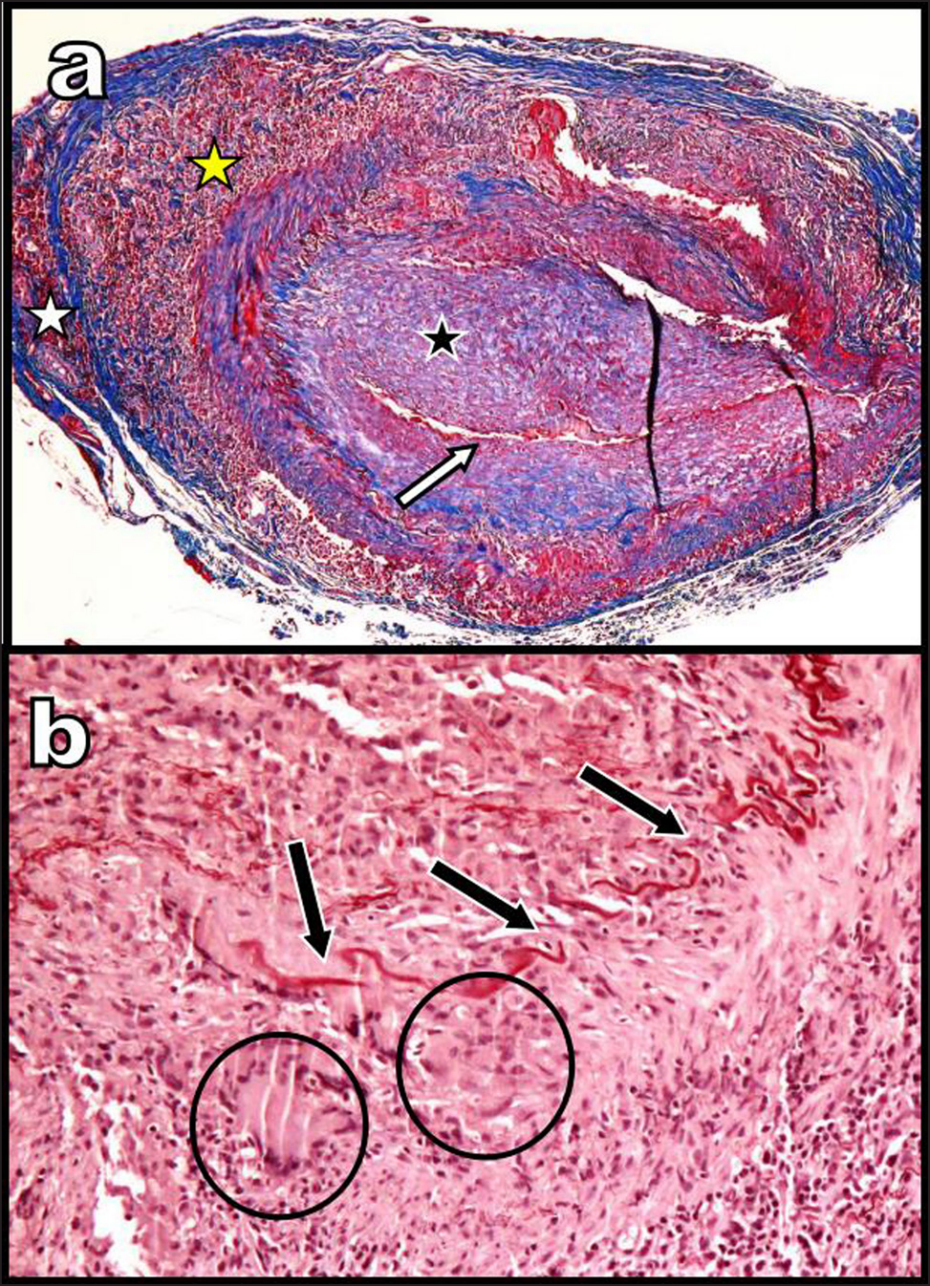
Histopathology of the temporal artery biopsy.

A 65-year-old woman presented with a three-month history of persistent ocular-nasal catarrh, sub-febrile state, myalgia, night sweats, weight loss and early daily bi-temporal pain. Severe biological inflammatory syndrome with CRP at 105 mg/l and sedimentation rate at 97 mm was present. Colour Doppler Ultrasound (CDU) (Figure 1) showed a typical inflammatory “halo sign” (black arrowhead on a) of the temporal arteries. A similar “halo sign” was found along the common carotid (white arrows on c, d, and e) and vertebral arteries (black arrows on d and e). The intima remained visible (white arrowheads on e). Computed Tomography (CT) angiography (Figure 2) showed blur homogeneously enhancing wall thickening of the aortic arch (white arrows on a and d) and of its large emerging arteries (white arrows on b). The axillary arteries were also affected (black arrows on a). Moderate thickening of the abdominal visceral aorta was also found (not illustrated). Hypodensity of the intima contrasted with enhancement of the inflamed media (white arrowheads on b). Temporal artery biopsy (Figure 3a and b) confirmed typical giant cell arteritis (GCA) with involvement of the media (yellow star) and adventice (white star) by chronic lymphocytic inflammation. Reactive intimal hyperplasia (black star) causing luminal collapse (white arrow), characteristic fragmentation of the internal elastic lamina (black arrows) and giant cells (black circles) were also diagnosed. Classical massive corticosteroid treatment was immediately started with rapid clinical and biological improvement. The “halo sign” and diffuse arterial thickening had, drastically, nearly completely resolved on post-therapeutic CDU (Figure 1b and f) and CT (Figure 2c and e) nine months later.

## Comment

Giant Cell Arteritis (GCA) is the most common large-vessel vasculitis, predominantly affecting females over 50 years of age and being frequently associated with Polymyalgia Rheumatica. It involves the aorta and/or its major branches but has a predilection for the extracranial branches of the carotid artery, especially the temporal artery [1].

There are three predominant clinical subtypes of GCA. The systemic inflammatory syndrome subtype presents with non-specific constitutional inflammatory symptoms. The cranial arteritis subtype manifests by headaches or facial pain, scalp tenderness, jaw claudication, painful dysphagia and vision loss. An inaugural transient visual loss is a true ophtalmological emergency. The large-vessel vasculitis type is characterized by subclavian and axillary arteries and/or aortic involvement. Aortic vasculitis may remain clinically silent but may secondarily lead to occult aneurysmal dilation.

Laboratory tests usually show a highly elevated erythrocyte sedimentation rate and C-reactive protein (CRP) level. Temporal artery biopsy remains the gold standard for diagnosis but CDU of temporal arteries is an alternative non-invasive, reproducible and inexpensive method of diagnosis. The “halo sign” is typical. Complementary cross-sectional imaging modalities comprising MDCT and/or MRI and PET scan are accurate in detecting the involvement of deep vessels.

A prompt initiation of Corticosteroid treatment generally gives rapid and spectacular improvement of symptoms but this does not prevent relapses.
